# Acetylation of the Pro-Apoptotic Factor, p53 in the Hippocampus following Cerebral Ischemia and Modulation by Estrogen

**DOI:** 10.1371/journal.pone.0027039

**Published:** 2011-10-26

**Authors:** Limor Raz, Quan-guang Zhang, Dong Han, Yan Dong, Liesl De Sevilla, Darrell W. Brann

**Affiliations:** Institute of Molecular Medicine and Genetics, Georgia Health Sciences University, Augusta, Georgia, United States of America; National Institute of Health, United States of America

## Abstract

**Background:**

Recent studies demonstrate that acetylation of the transcription factor, p53 on lysine^373^ leads to its enhanced stabilization/activity and increased susceptibility of cells to stress. However, it is not known whether acetylation of p53 is altered in the hippocampus following global cerebral ischemia (GCI) or is regulated by the hormone, 17β-estradiol (17β−E_2_), and thus, this study examined these issues.

**Methodology/Principal Findings:**

The study revealed that Acetyl p53-Lysine^373^ levels were markedly increased in the hippocampal CA1 region after GCI at 3 h, 6 h and 24 h after reperfusion, an effect strongly attenuated by 17β−E_2_. 17β−E_2_ also enhanced interaction of p53 with the ubiquitin ligase, Mdm2, increased ubiquitination of p53, and induced its down-regulation, as well as attenuated elevation of the p53 transcriptional target, Puma. We also observed enhanced acetylation of p53 at a different lysine (Lys^382^) at 3 h after reperfusion, and 17β−E_2_ also markedly attenuated this effect. Furthermore, administration of an inhibitor of CBP/p300 acetyltransferase, which acetylates p53, was strongly neuroprotective of the CA1 region following GCI. In long-term estrogen deprived (LTED) animals, the ability of 17β−E_2_ to attenuate p53 acetylation was lost, and intriguingly, Acetyl p53-Lysine^373^ levels were markedly elevated in sham (non-ischemic) LTED animals. Finally, intracerebroventricular injections of Gp91ds-Tat, a specific NADPH oxidase (NOX2) inhibitor, but not the scrambled tat peptide control (Sc-Tat), attenuated acetylation of p53 and reduced levels of Puma following GCI.

**Conclusions/Significance:**

The studies demonstrate that p53 undergoes enhanced acetylation in the hippocampal CA1 region following global cerebral ischemia, and that the neuroprotective agent, 17β−E_2_, markedly attenuates the ischemia-induced p53 acetylation. Furthermore, following LTED, the suppressive effect of 17β−E_2_ on p53 acetylation is lost, and p53 acetylation increases in the hippocampus, which may explain previous reports of increased sensitivity of the hippocampus to ischemic stress following LTED.

## Introduction

Stroke is the third leading cause of death and the number one cause of disability in the United States [1]–[3]. In order to help understand the molecular mechanisms and processes that underlie neuronal cell death following ischemic stroke, animal models of focal and global cerebral ischemia (GCI) have been developed [4], [5] [6], [7]. The hippocampal CA1 region, an area critical for learning and memory [8]–[10], is highly sensitive to damage following GCI. Along these lines, neurons in the hippocampal CA1 region have been shown to undergo delayed apoptotic neuronal cell death following GCI [8], [9], [11]. Apoptotic neuronal cell death has also been shown to occur in the penumbra region of the cerebral cortex after focal cerebral ischemia [12].

P53, a pro-apoptotic factor, has been implicated to play a major role in apoptotic neuronal cell death following cerebral ischemia [13]. Evidence from p53 knock-out mice studies revealed reduced neuronal cell death after GCI as compared to WT-controls [14]. In addition, pifithrin-alpha (PFTα), a p53 specific pharmacological inhibitor, attenuated p53 nuclear transport and DNA binding, while increasing the number of surviving neurons [15] and promoting functional recovery following stroke [16]. In non-neuronal cells, p53 has been implicated to induce cell death following cell stress via both a transcriptional-dependent nuclear mechanism, as well as a transcription-independent mechanism involving its direct action with a subset of Bcl-2 family member proteins in the cytosol and mitochondria. However, recent work suggests that the apoptotic activity of p53 in neurons does not rely on its direct action at the cytosol/mitochondria and appears to be mediated exclusively through its transcription-dependent nuclear functions to induce the p53 pro-apototic BH3 family gene, Puma (p53 upregulated modulator of apoptosis) [17].

Noxa (Latin for damage) is another BH3 family pro-apoptotic gene that is induced by p53 transcriptional activity [18], [19]. Puma and Noxa have been implicated to directly and indirectly activate Bax (Bcl-2-associated X protein) and Bak (Bcl-2-antagonist/killer), resulting in permeabilization of the outer mitochondrial membrane, release of cytochrome c and induction of apoptosis [20], [21]. Although both Puma and Noxa have been shown to mediate neuronal cell death, only Puma deficiency was significantly protective against apoptosis, stressing the importance of this factor in the apoptotic cascade [22]–[24]. With respect to cerebral ischemia, work by Chan and coworkers [25] demonstrated that cerebral ischemia induced Puma upregulation in the hippocampal CA1 region was inhibited by the p53 inhibitor, PFTα, which was correlated with significant neuroprotection.

Due to its critical role in inducing apoptotic cell death, there has been intense study on the mechanisms of regulating p53 stability and activation, including post-translational modifications. Along these lines, recent work in cancer cells has shown that p53 activity and stability can be enhanced by acetylation at the Lysine^373^ residue (and possibly Lysine^382^ residue) on the C-terminal of the p53 protein, which leads to enhanced susceptibility of cells to stress [26]–[30]. This activation and acetylation of p53 is achieved by the CBP/p300 family of acetyl transferases [31]–[33]. A role for p53 acetylation in enhancing apoptosis of cortical neurons *in vitro* after exposure to various cell stressors has also been reported [34], [35]. However, it is currently unknown whether p53 acetylation state is altered in the brain *in vivo* after cerebral ischemia. Since a change in the acetylation state of p53 following cerebral ischemia could affect its stability and activity and influence neuronal susceptibility to ischemic stress, a major goal of the current study was to address this key deficit in our knowledge.

Previous work by our group and others has shown that the steroid hormone, 17β−Estradiol (17β−E_2_) can exert potent anti-apoptotic and neuroprotective effects in the hippocampal CA1 region following GCI, in part through an anti-oxidant effect to attenuate activation of NADPH oxidase and ROS generation [36], [37]. Thus, an additional goal of our studies was to determine whether 17β−E_2_ and NADPH oxidase play a critical role in regulating p53 acetylation and corresponding induction of p53-regulated factors in the hippocampus following cerebral ischemia.

## Methods

### Induction of Global Cerebral Ischemia

Adult Sprague-Dawley female rats (3 months of age) were bilaterally ovariectomized (OVX) and immediately implanted with placebo (Pla) or 17β-estradiol (17β−E_2_) Alzet minipumps (0.025 mg; 14–21 day release), producing a low physiological diestrus I level of 10–15 pg/ml of 17β−E_2_ in the blood [10]. In some animals, long-term 17β−E_2_ deprivation was performed in which the animals were ovariectomized and 10 weeks later (*10* *W*), placebo or 17β−E_2_ minipumps were implanted as previously described [36]. A 4-vessel occlusion (4-VO) model of global cerebral ischemia (GCI) was performed one week after initiation of 17β−E_2_ replacement, as described previously [36], [38]. GCI is a well known model of delayed neuronal cell death in which hippocampal CA1 pyramidal neurons are highly susceptible to damage and cell death [11]. Briefly, for induction of GCI, animals were first anesthetized with chloral hydrate (350 mg/kg, i.p.). The vertebral arteries were then electrocauterized and the common carotid arteries (CCA) were exposed. Wound clips were then used to close the incision and the rats were allowed a 24 h recovery period. Following 24 h, the animals were anesthetized using 3% isoflurane anesthesia and the CCA were re-exposed and clipped via aneurysm clips. GCI occlusion was performed for 10 min. duration. Rats which lost their righting reflex within 30 sec. and whose pupils were dilated and unresponsive to light during ischemia were selected for the experiments. Carotid artery blood flow was restored by releasing the clips and allowing reperfusion. Rectal temperature was maintained at 37°C using a thermal blanket throughout the experiment and 2 h thereafter. Sham controls underwent the same surgical exposure procedures, except that the arteries were not occluded. All procedures were approved by the Georgia Health Science University institutional committee for care and use of animals (AUP# 09-03-174) and were in accordance with National Institutes of Health guidelines.

### Drug Administration

To determine whether crosstalk occurs between NADPH oxidase-induced membrane superoxide (O_2_^−^) production and downstream pro-apoptotic factors, Gp91ds-Tat, a competitive NOX2 inhibitor and its scrambled peptide control were used. Gp91ds-Tat is a competitive inhibitor consisting of a 9 amino-acid peptide sequence of the p47phox subunit docking site on NOX2, thereby preventing p47phox from forming a complex with NOX2 and activating the enzyme [39]. Gp91ds-Tat and the scrambled peptide control were administered into the lateral ventricles of the hippocampus CA1, at a dose of 100 ng in 5 µl. The dose of gp91ds-Tat was chosen based on previous studies by our laboratory showing its effectiveness in inhibiting NADPH oxidase and lack of side effects [36]. For intracerebroventricular (icv) injections, anesthetized rats were placed on ear bars of a stereotaxic instrument. Drug infusion was performed using a stepper motorized microsyringe (Stoelting, Wood Dale, IL, USA) at a rate of 1 µL/min into the cerebral ventricle (from the bregma: anteroposterior,±0.8 mm; lateral, 1.5 mm; depth, 3.5 mm). When necessary, curcumin (200 mg/kg, sc-294110, Santa Cruz Biotechnology, Inc.) or DMSO vehicle was injected intraperitoneally, 30 min prior to the induction of GCI.

### DAB Staining

Sections for DAB staining were incubated with 10% normal donkey serum in PBS containing 0.1% Triton X-100 and 0.3% H_2_O_2_ to block nonspecific surfaces for 1 h at room temperature. Sections were then incubated with the primary antibodies overnight at 4° Celsius (C°) in PBS containing 0.1% Triton X-100. The antibodies used were as follows: goat anti-Noxa (1∶200, Santa Cruz Biotechnology, Santa Cruz, CA) and rabbit anti-Acetyl p53-lysine^373^ (1∶200, Millipore, Billerica, MA). Afterwards, sections were washed with the same buffer, followed by incubation with a secondary biotinylated anti-goat or rabbit antibodies (Molecular Probes, Eugene, OR, USA) at a dilution of 1∶500 in PBS containing 0.1% Triton X-100 for 1 h at room temperature. Sections were then washed, followed by incubation with ABC reagents for 1 h at room temperature in the same buffer. Sections were rinsed with the same buffer and incubated with DAB reagent for 2–10 min., according to the instructions of the manufacturer (Vector Laboratories). Sections were briefly washed with distilled water and dehydrated in graded alcohols, cleared in xylene, and mounted using xylene-based mounting medium following DAB staining. Images were captured on an Axiophot-2 visible microscope using an AxioVision4Ac software system (Carl Zeiss, Germany) at a magnification of 10X or 40X, respectively. The number of NOXA or Acetyl p53-lysine^373^-positive cells was counted, as described previously [36]. Briefly, for quantitative analyses, neurons which stained positively for Noxa or acetylated p53 were counted bilaterally per 250 µm length of medial CA1 pyramidal cell layer, in four to five sections per animal to provide a single value for each animal. A mean±SE was calculated from the data in each group (*n* = 5–6 animals), and statistical analysis was performed as described below.

### Double Immunofluorescence Staining

Coronal sections were incubated with 10% normal donkey serum for 1 h at room temperature in PBS containing 0.1% Triton X-100, followed by incubation with appropriate primary antibodies overnight at 4°C in the same buffer. The following primary antibodies were used in different combinations: rabbit anti-acetyl p53-Lysine^373^, mouse anti-NeuN (1∶200, Millipore, Billerica, MA) and rabbit anti-Puma α/β (1∶200, Santa Cruz Biotechnology, Santa Cruz, CA). After primary antibody incubation, sections were washed and incubated with Alexa Fluor594/647 donkey anti-rabbit or Alexa-Fluor488/594 donkey anti-mouse secondary antibodies (1∶500; Invitrogen Corporation, Carlsbad, CA), respectively, for a period of 1 h at room temperature. Sections were washed with buffer, followed by PBS and then briefly, with water. Sections were mounted using water-based mounting medium with anti-fading agents (Biomeda, Fisher Scientific, Pittsburgh, PA). Negative experimental controls were incorporated by omission of the primary antibody, thus confirming the absence of non-specific immunofluorescent staining, cross-immunostaining, or fluorescence bleed-through.

### Confocal Microscopy and Image Analysis

Double-labeled images were captured on an LSM510 Meta confocal laser microscope (Carl Zeiss, Germany) using a 40X oil-immersion Neofluor objective with a 1.3 numerical aperture. The image size was set at 1024×1024 pixels, as previously published [40]. Excitation/emission laser filters settings were used for various chromophores as follows: argon/2 laser was used for Alexa Fluor488, with excitation maximum at 490 nm and emission in the range of 505–530 nm and HeNe1 laser was used for Alexa Fluor594, with excitation maximum at 543 nm and emission in the range of 568–615 nm. The captured images were viewed and analyzed using LSM510 Meta imaging software.

### Brain Homogenates and Subcellular Fractionations

For brain tissue preparation, rats were sacrificed under isoflurane anesthesia after GCI, at the timepoints described in the figure legends. The CA1 region of the hippocampus was micro-dissected bilaterally from the hippocampal fissure and immediately frozen in liquid nitrogen. The protocol for obtaining total tissue lysates was as follows: tissue homogenization was performed with a Teflon-glass homogenizer in ice cold homogenization medium consisting of 50 mM HEPES (pH 7.4), 150 mM NaCl, 12 mM β-glycerophosphate, 3 mM dithiotheitol (DTT), 2 mM sodium orthovanadate (Na3VO4), 1 mM EGTA, 1 mM NaF, 1 mM phenylmethylsulfonyl fluoride (PMSF), 1% Triton X-100, and 10 µg/ml each of aprotinin, leupeptin, and pepstatin A. The homogenates were centrifuged at 15,000×g for 30 min. at 4°C and total lysate supernatants were collected and stored at −80°C for later use. Protein concentrations were determined by a Lowry protein assay kit with bovine serum albumin utilized as a standard.

### Co-Immunoprecipitation

For co-immunoprecipitation (Co-IP), total fractions (each containing 200 µg of protein) obtained at the ischemic reperfusion timepoints described in the figure legends, were diluted 4-folds with 500 µL of HEPES buffer containing the following reagents: 50 mM HEPES (pH7.4), 150 mM NaCl, 10% glycerol, 1% Triton X-100, and 1 mM each of EGTA, EDTA, PMSF and Na_3_VO_4_. The samples were then pre-incubated with 20 µl protein A/G and centrifuged to remove non-specific protein bound to the A/G protein. The supernatant was incubated with 5 µg of mouse anti-p53 antibody (Santa Cruz Biotechnology Inc., Santa Cruz, CA) and mixed for 4 h at 4°C. After the addition of Protein A/G-sepharose, the mixture was incubated at 4°C for 2 additional hours. Samples were washed three times with HEPES buffer and eluted by sodium dodecyl sulfate-polyacrylamide gel electrophoresis (SDS-PAGE) loading buffer, then boiled for 5 min.

### Western Blots

Western blotting was performed on total lysates collected post-reperfusion, as described in the relevant figure legends and in our laboratory publications [10]. The antibodies used were as follows: Rabbit anti-Acetyl p53 specifically recognizing p53 acetylation at Lysine^373^ by p300, (1∶150, Millipore, Billerica, MA), Rabbit anti-Acetyl-p53 (Lys^382^, 1∶200, Cell Signaling Technology, Inc.), Rabbit anti-Actin (1∶300, Sigma-Aldrich, St. Louis, MO), rabbit anti-Mdm2 (1∶200, Santa Cruz Biotechnology, Santa Cruz, CA), rabbit anti-p53 (1∶200, Cell Signaling Technology, Inc., MA), rabbit anti-Puma α/β (1∶150, Santa Cruz Biotechnology, Santa Cruz, CA) and mouse anti-Ubiquitin (Santa Cruz Biotechnology, Santa Cruz, CA). The membrane was then washed with T-TBS to remove unbound antibody, followed by incubation with Alexa Fluor 680 goat anti-rabbit/mouse IgG and Alexa Fluor 680 donkey anti-rabbit/goat IgG for 1–2 h at room temperature. Bound proteins were visualized using the Odyssey Imaging System (LI-COR Bioscience, Lincoln, NB) and semi-quantitative analysis of the bands was performed with Image J analysis software (Version 1.30 v; NIH, USA). Band densities for the indicated proteins were normalized and expressed relative to actin house-keeping gene total protein or total p53 protein, as indicated in the figures. Normalized means were then expressed as fold change of the corresponding value for control (sham operated) animals. A Mean±SE was calculated from the data, from 4–5 animals per treatment group for graphical presentation and statistical comparison.

### Real Time-PCR

Total RNA from the female rat hippocampal CA1 region was isolated following ischemic reperfusion as described in the corresponding figure legends and LightCycler RNA Amplification Kit SYBR Green I (Roche Applied Science, IN) was utilized. A negative control, which consisted of pooled total RNA run in the RT-PCR without RT added, was also included. Actin was used as a loading control. Five hundred nanograms of the RT reactions were used for the PCR, with the following primer sets to identify p53: forward primer 5’- TCT CCC CAG CAA AAG AAA AA - 3’ and reverse primer 5’ – CTT CGG GTA GCT GGA GTG AG – 3’. PCR reactions were conducted under the following conditions: 35 cycles at 95°C for 5 min, followed by 65°C for 30 min and 72°C for 3 min. at an annealing temperature of 65°C and 4 mM of MgCl_2_. A total of 5 µL of the PCR product was electrophoresed, visualized with ethidium bromide, and photographed for documentation. A standard curve was generated and results were quantified accordingly in ρg/mL.

### Statistical Evaluation

Four to five animals were used per treatment group. All values were expressed as the means±SE. Statistical analysis of the results was carried out by One-Way Analysis of Variance (ANOVA), followed by the Student-Newman–Keuls post-hoc test to determine group differences. When groups were compared to a control group (e.g. sham), Dunnett's test was adopted for post-hoc analyses after ANOVA. Statistical significance was accepted at the 95% confidence level (p<0.05). Data was expressed as mean±standard error (SE).

## Results

### 17β−E_2_ Attenuates Cerebral Ischemia-Induced Elevation of p53 and Acetyl p53-Lysine^373^ Levels in the Hippocampal CA1 Region

**Figure 1:**
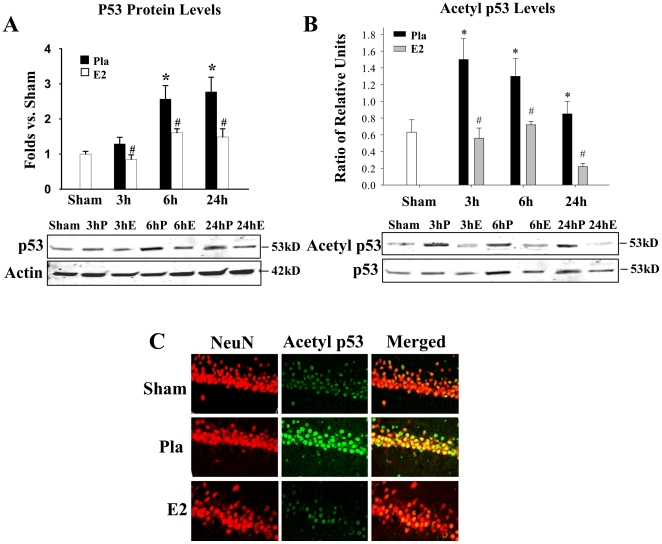
P53 levels and acetylation of p53 on Lysine^373^ are increased in the hippocampus following global cerebral ischemia and attenuated by 17β-estradiol. (A & B) Homogenates obtained from the hippocampus CA1 at 3 h, 6 h and 24 h after reperfusion were subjected to Western blot analyses to examine the temporal expression of p53 (A) and Acetyl p53-Lys^373^ (B). Actin bands represent loading control. Data is expressed as fold differences as compared to sham (A) and Acetyl p53/p53 protein ratio expressed in relative units (B) from four to five animals. *p<0.05 vs. Sham, #*p*<0.05 vs. Pla control; S = Sham, P = Pla, E = E_2_. (C) Representative hippocampal CA1 sections were double labeled with the neuronal marker NeuN (red) and Acetyl p53 (green) at 24 h following ischemic reperfusion. Merged images represent acetylated p53 in pyramidal CA1 neurons of the hippocampus. Magnification 40X. n = 4–5 animals per treatment group.

**Figure 1A** shows that p53 levels in the hippocampal CA1 region are significantly increased at 6 h and 1 d after cerebral ischemia (Pla) as compared to sham controls. P53 levels at 3 h after reperfusion showed a pattern for elevation, which, however, was not statistically significant. In addition, 17β−E_2_ treatment significantly attenuated p53 elevation at 3 h, 6 h and 24 h after ischemic reperfusion (**Fig. 1A**). **Figure 1B** shows Acetyl p53-Lysine^373^ levels (expressed as ratio of Acetyl p53-Lysine^373^/p53) in the hippocampal CA1 region at various time-points after GCI. The data shows that Acetyl p53-Lysine^373^ levels increase significantly in the hippocampal CA1 region at all time-points examined after GCI (3 h, 6 h and 24 h) as compared to sham controls. In addition, 17β−E_2_ treatment significantly attenuated ischemia-induced Acetyl p53-Lysine^373^ levels at all time-points after GCI reperfusion, with the most robust effect seen at 3 h following ischemic reperfusion (**Fig. 1B**). To determine in which cell types acetylation of p53 occurred, we performed double immunohistochemistry on hippocampal CA1 sections collected at 24 h after GCI for the neuronal marker, NeuN (red), and Acetyl p53-Lysine^373^ (green) **(** **Fig. 1C**). The results show that Acetyl p53-Lysine^373^ staining is highly co-localized in neurons, and confirm the ischemic reperfusion elevation of Acetyl p53-Lysine^373^ levels in the CA1 region after cerebral ischemia (Pla vs. Sham), and the profound attenuation of p53 acetylation by 17β−E_2_ (E2), as compared to placebo-treated (Pla) controls at 24 h following cerebral ischemia **(** **Fig. 1C**). It should be mentioned that the regulatory effect of 17β−E_2_ on acetylation of p53 at Lys^373^ was only observed in animals following elevation by cerebral ischemia, as we observed no regulation by 17β−E_2_ of basal Acetyl p53-Lysine^373^ levels in non-ischemic sham controls (**Figure S1A**).

### Estrogen attenuates p53 acetylation at Lysine^382^ shortly after ischemic reperfusion

Previous studies have shown that p53 can also be acetylated on Lysine ^382^, and that this increases stability of p53 [41]. We therefore examined whether Acetyl p53-Lysine^382^ levels increase in the hippocampal CA1 region at 3 h after GCI (the time when Acetyl p53-Lysine^373^ displayed peak levels) and determined whether 17β−E_2_ similarly attenuates acetylation of p53 at the Lys^382^ site. The results of the study revealed a marked increase in Acetyl p53-Lysine^382^ levels in the hippocampal CA1 region of placebo (Pla)-treated animals at 3 h after GCI as compared to sham controls. In addition, 17β−E_2_ significantly attenuated the elevation of Acetyl p53-Lysine^382^ levels (**Figure S1B**). Note that total p53 levels remained unchanged in all treatment groups examined, and that 17β−E_2_ had no effect on basal Acetyl p53-Lysine^382^ levels in non-ischemic sham animals.

### 17β-Estradiol Down-Regulates BH3 Family Members Puma and Noxa Following Ischemia

**Figure 2:**
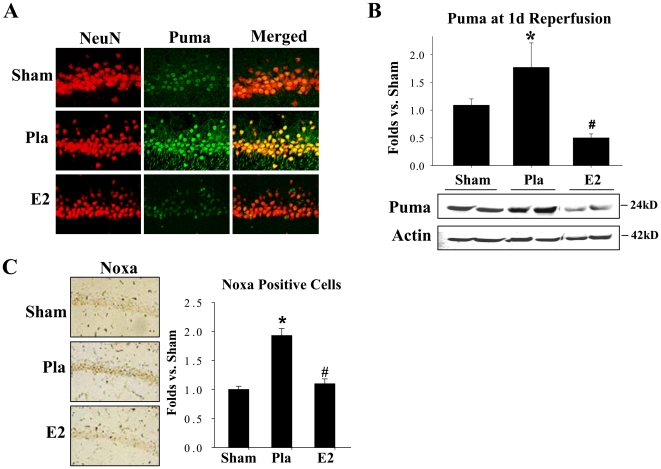
17β-Estradiol attenuates downstream pro-apoptotic BH3 family members, Puma and Noxa, in the CA1 following ischemia. (A) Hippocampal CA1 sections were double stained for NeuN neuronal marker (red) and Puma (green) at 24 h following global cerebral ischemia. Merged images depict Puma neuronal localization in the CA1 region of the hippocampus. Magnification 40X. (B) Western blot using anti-Puma antibody shows Puma protein expression at 24 h following ischemic reperfusion. Actin bands represent loading control. Data is expressed as fold differences in comparison to sham from four to five animals. *p<0.05 vs. Sham, ^#^ *p*<0.05 vs. Pla; Pla = Placebo, E2 = 17β−E_2_. (C) Effect of 17β−E_2_ treatment on Noxa protein expression at 24 h reperfusion timepoint following cerebral ischemia. Representative photomicrographs of DAB staining obtained at a magnification of 10X (left panels). Semi-quantitative analysis of Noxa staining expressed as the number of Noxa-positive cells per 250 µm area obtained from 4–5 hippocampal sections per animal (right panels). *p<0.05 vs. Sham, ^#^ *p*<0.05 vs. Pla. n = 4–5 animals per treatment group.

We next examined the expression of the downstream p53 transcriptional target proteins, Puma and Noxa. In addition, we performed double immunohistochemistry on coronal hippocampal CA1 sections from the 24 h time-point for the neuronal marker, NeuN, (red) and Puma (green) to determine whether Puma exhibits neuronal localization and is regulated by 17β−E_2_. As shown in **Fig. 2A**, representative images revealed Puma predominantly localized in pyramidal neurons of the hippocampus CA1 region in all treatment groups studied. Of significant interest, the results revealed a robust elevation of Puma immunoreactive staining in placebo (Pla) animals following GCI as compared to sham controls, and 17β−E_2_ (E2) treatment strongly attenuated this elevation. **Figure 2B** shows Western blot analysis of total Puma protein levels in hippocampal CA1 region samples collected at 24 h after GCI. As shown in **Fig. 2B**, similar to the immunostaining results, Western blot analysis revealed that Puma levels are significantly elevated in placebo (Pla) animals after GCI as compared to sham controls, and that 17β−E_2_ (E2) treatment significantly attenuated the elevation of Puma. In **Figure 2C**, we examined an additional p53-induced BH3 family pro-apoptotic protein, Noxa, via DAB immunostaining. As shown in **Fig. 2C** **(left panel)**, representative photomicrographs of Noxa immunostaining reveal a similar pattern to that of Puma up-regulation in ischemic animals, and 17β−E_2_ treatment markedly attenuating the ischemia-induced up-regulation of Noxa. Semi-quantitative analysis of the Noxa staining results through counting of the number of Noxa-positive cells in the hippocampal CA1 sections confirmed an elevation of Noxa-positive cells in the placebo (Pla) group as compared to the sham controls, and a strong attenuation of the number of Noxa-positive cells by 17β−E_2_ (E2) (**Fig. 2C, right panel**).

### 17β−E_2_ Enhances Both the Interaction of the Ubiquitin Ligase, Mdm2 with p53 and the Ubiquitination of p53 Following GCI

**Figure 3:**
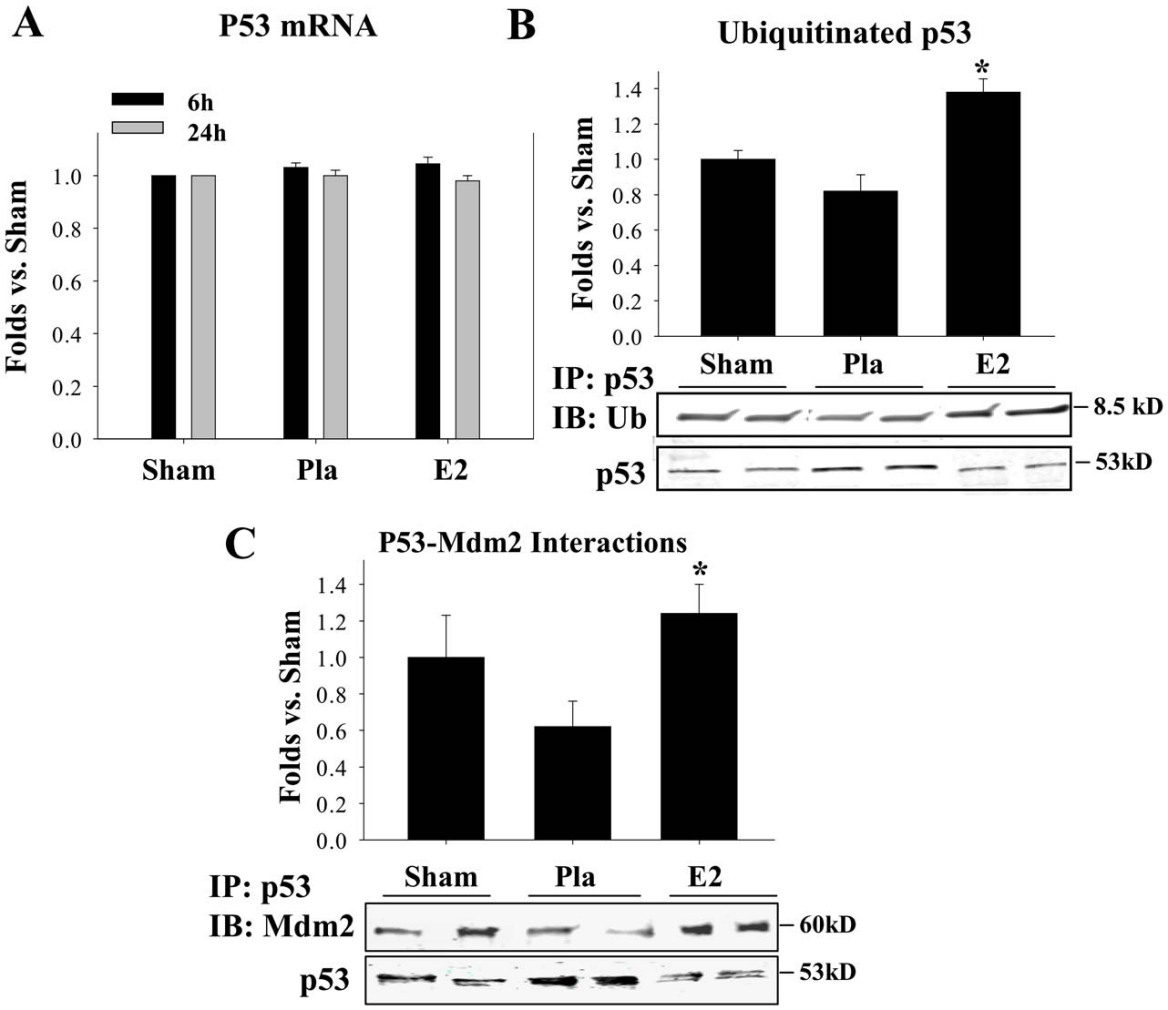
17β-Estradiol enhances p53 interaction with Mdm2 and p53 ubiquitination. (**A**) P53 mRNA levels remain unchanged in the hippocampus CA1 at 6 h and 24 h following cerebral ischemia as measured by real-time RT-PCR. (**B**) 17β−E_2_ increases p53-ubiquitin interactions and decreases p53 levels at 24 h after GCI reperfusion. *p<0.05 vs. Pla. **(C)** 17β−E_2_ increases p53-Mdm2 binding two fold as compared to placebo-treated control levels at 6 h after cerebral ischemic injury. P53 protein expression level serves as Co-IP control. *p<0.05 vs. Pla. Pla = Placebo, E2 = 17β−E_2_._._ n = 4–5 animals per treatment group.

It has been reported that deacetylation of p53 at Lysine^373^ decreases the stability of p53 and leads to its ubiquitination and degradation [42], [43]. The E3 ubiquitin ligase, Mdm2, has been implicated to be an important factor responsible for ubiquitination of p53 [44], [45]. We thus examined whether Mdm2 interaction with p53 and ubiquitination of p53 in the hippocampal CA1 region is modulated by 17β−E_2_ following GCI. We also examined p53 mRNA levels to rule out that p53 changes after GCI or 17β−E_2_ treatment were due to changes in p53 gene expression. **Figure 3A** illustrates the results of examination of p53 gene expression in the hippocampal CA1 region at 6 h and 1 d after GCI using real time (RT)-PCR. As shown in **Fig. 3A**, neither ischemia (Pla) nor 17β−E_2_ (E_2_) treatment significantly affected p53 gene expression in the hippocampal CA1 region. Actin, used as a loading experimental control, revealed equal loading on the RT-PCR gel (data not shown). We next examined ubiquitination of p53 by co-immunoprecipitated (Co-IP) of p53 and immunoblotting for ubiquitin at the 24 h time-point after reperfusion using total hippocampal CA1 region samples. The results showed that 17β−E_2_ treatment significantly increases p53 ubiquitination as compared to placebo-treated or sham control animals (**Fig. 3B**). Co-IPs were also performed examining p53-Mdm2 interaction in the hippocampal CA1 region after GCI (**Fig. 3C**). As shown in **Fig. 3C**, the Pla group had a pattern for a decrease of p53-Mdm2 interaction that correlated with an increase of p53 protein levels. In contrast, 17β−E_2_-treated animals had a significant enhancement of p53-Mdm2 interaction in the CA1 region, which correlated with a significant decrease in p53 protein levels (**Fig. 3C**).

### NOX2 NADPH Oxidase Inhibition Attenuates Acetyl p53/p53 Ratio and Puma Levels after GCI

**Figure 4:**
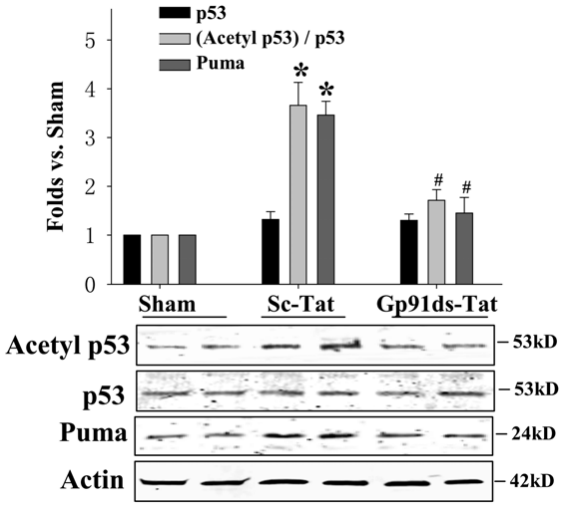
NADPH oxidase inhibition attenuates Acetyl p53-Lysine^373^ levels **and Puma levels following GCI.** Inhibition of NADPH oxidase using the competitive NOX2 inhibitor, Gp91ds-Tat (Gp91), reduces Acetyl p53-Lysine^373^ levels, while diminishing Puma protein levels, as compared to the scrambled tat (Sc-Tat) control group. CA1 total lysates were collected at 3 h upon reperfusion onset and probed with appropriate antibodies to detect protein expression in Western blot. P53 protein expression remained unchanged. Actin was used as a loading control. Data is represented as fold differences in comparison to sham from four to five animals. *p<0.05 vs. Sham, #*p*<0.05 vs. Sc-Tat; S = Sham.

Previous work by our laboratory revealed that 17β−E_2_ can attenuate NOX2 NADPH oxidase-induced superoxide (O_2_^−^) production, and that administration of a competitive NOX2 inhibitor, Gp91ds-Tat, is strongly neuroprotective against GCI-induced neuronal damage in the hippocampal CA1 region [36]. We therefore hypothesized that NADPH oxidase-induced ROS may regulate p53 and its acetylation following GCI. To address this possibility, we used icv injections of the specific NOX2 inhibitor, Gp91ds-Tat. As a control, we used a scrambled tat peptide (Sc-Tat) that does not exert inhibitory effects on NADPH oxidase. Pro-apoptotic p53 levels remained unchanged in all of the treatment groups examined, further validating our previous observations (**Figure 4**). When compared to scrambled-tat control peptide-treated animals, Gp91ds-Tat-treated animals had a significant attenuation of GCI-induced elevation of Acetyl p53 (expressed as an acetyl p53/p53 ratio) and a significant decrease in Puma protein levels in the hippocampal CA1 region at 3 h after GCI reperfusion, a time of peak NADPH oxidase activation following GCI [36]. This finding suggests that NADPH oxidase activation potentially contributes to cerebral ischemia-induced elevation of acetyl p53 and downstream Puma expression following GCI (**Figure 4**).

### Long-term Estrogen Deprivation Promotes p53 Acetylation and Activation in the Nucleus of Hippocampal CA1 Cells after Global Cerebral Ischemia

**Figure 5:**
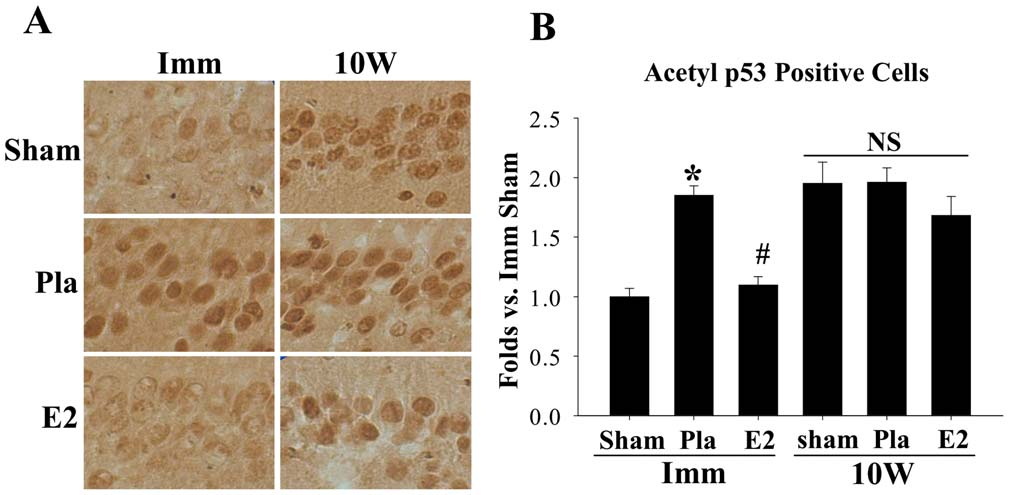
The ability of estrogen to suppress the elevation of Acetyl p53-Lysine^373^ levels following GCI is lost in long-term estrogen deprived animals. (A) Representative DAB images of immediate (*Imm*, 7 d) and 10 week (*10* *W*) E2 deprived animals at 24 h following ischemic reperfusion. Note that 17β−E_2_ attenuation of Acetyl p53-Lysine^373^ levels is lost in long-term E2 deprived (*10* *W*) animals and that there is robust p53 acetylation in all *10* *W* animals. Magnification 40X. (B) Semi-quantitative analysis of Acetyl p53-Lysine^373^-positively stained cells in *Imm* (7 d) and long-term (*10* *W*) E_2_-deprived animals, expressed as folds vs. sham in 4–5 sections. *p<0.05 vs. Sham, #*p*<0.05 vs. Pla or 10 W E2 group; NS = No significant difference. n = 4–5 animals per treatment group.

Finally, our group recently demonstrated that 17β−E_2_ neuroprotection against GCI is lost if it is preceded by a prolonged period of 17β−E_2_ deprivation (e.g. 10 weeks ovariectomy) [36]. We thus examined whether long-term estrogen deprivation leads to a loss of 17β−E_2_ ability to attenuate Acetyl p53-lysine^373^ levels in the hippocampal CA1 region after GCI. We therefore compared animals treated immediately with 17β−E_2_ (*Imm*) to those in which 17β−E_2_ treatment was initiated after a long term period of 17β−E_2_ deprivation (e.g. treated 10 weeks after ovariectomy) (*10* *W*) (**Figure 5A, 5B**). Representative photomicrographs of the hippocampal CA region from the 24 h time-point after GCI from the various groups are presented in **Fig. 5A**, and semi-quantitative analysis of number of Acetyl p53-lysine^373^ positive cells are presented in **Fig. 5B**. As shown in **Fig 5A, 5B**, in the immediate (*Imm*) treatment paradigm, Pla-treated animals had robust staining and number of Acetyl p53-lysine^373^ positive cells in the CA1 region as compared to sham controls, while immediate 17β−E_2_-treatment caused a profound reduction in the staining and number of Acetyl p53-lysine^373^ positive cells. In contrast, in long-term E_2_ deprived (10 W) sham animals, there was robust Acetyl p53 staining and a high number of Acetyl p53-lysine^373^-positive cells (**Fig. 5A, 5B**). In addition, long-term E2 deprived (*10* *W*) *Pla* animals also showed robust Acetyl p53 staining and a high number of Acetyl p53-lysine^373^ positive cells in the hippocampal CA1 region, which was not significantly different from the *10* *W* sham controls. Interestingly, the ability of 17β−E_2_ to attenuate Acetyl p53 staining and number of Acetyl p53-lysine^373^-positive cells was completely lost in the long-term E_2_ deprived animals (**Fig. 5A, 5B**).

#### Inhibition of CBP/p300 acetyltransferase is neuroprotective of the hippocampus CA1 following global cerebral ischemia

**Figure 6:**
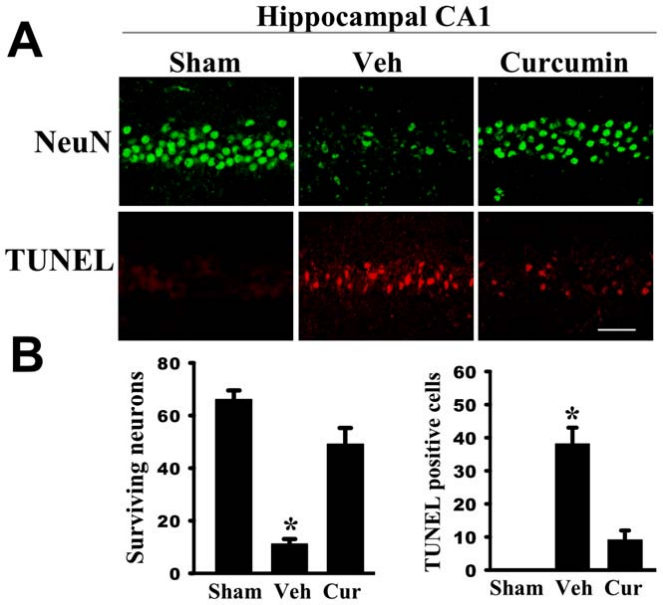
Neuroprotective effects of curcumin in the hippocampus CA1 following global ischemia. *(* ***A*** *)* Representative photomicrographs of the hippocampal CA1 region showing NeuN and TUNEL staining following 7 days of ischemic reperfusion. *(* ***B*** *)* Quantitative summary of data (means±SE, n = 6 animals per group) indicating the number of surviving neurons and apoptotic neurons per 250 µm length of medial CA1. NeuN positive pyramidal cells containing intact and round nuclei were counted as surviving cells. Scale bar = 50 µm, magnification 40X. *p<0.01 vs. sham and curcumin groups. Veh = vehicle; Cur = curcumin.

We next examined whether inhibition of CBP/p300 acetyltranferases, which acetylate p53, would exert neuroprotection against GCI. To examine this question, we utilized the selective CBP/p300 acetyltransferase inhibitor, curcumin [31], [32]. As shown in **Figure 6A and 6B**, administration of curcumin 20 minutes prior to GCI was strongly neuroprotective in the hippocampal CA1 region, as evidenced by preservation of NeuN-stained surviving cells and a decrease in TUNEL-positive cells in the CA1 region at 7 days after GCI.

## Discussion

The current study advances the field by demonstrating that p53 undergoes enhanced acetylation on Lysine^373^ and Lysine^382^ in the hippocampal CA1 region following global cerebral ischemia. Acetylation of p53 at Lysine^373^ and Lysine^382^ has been shown to enhance stability and pro-apoptotic activity of p53, while increasing sensitivity to cell stress [27]–[30], [41], [46]. Thus, our study suggests that post-translational modification of p53 by acetylation may be an important *novel* regulatory mechanism for enhancing p53 stability and activation following cerebral ischemia, leading to enhanced apoptotic cell death. Our study also shows that 17β−E_2_ acts in the hippocampus to *decrease* acetylation of p53 on Lysine^373^ and Lysine^382^ following GCI.

It is currently unclear how 17β−E_2_ is able to attenuate acetylation of p53. It is possible that the regulatory effects of 17β−E_2_ are mediated indirectly, through its previously reported ability to suppress NOX2 NADPH oxidase activation [36]. Along these lines, administration of a NOX2 inhibitor in the current study led to inhibition of p53 acetylation following GCI. This suggests that NOX2 activation may contribute to cerebral ischemia-induced p53 acetylation in the brain, potentially via induction of ROS. NADPH oxidase activation leads to generation of superoxide, which can be converted to highly reactive and damaging ROS such as hydroxyl ion and peroxynitrite and contribute to oxidative stress [47]. Intriguingly, a role for oxidative stress in regulating p53 acetylation is supported by previous *in vitro* studies which showed that oxidative stress markedly enhances p53 acetylation [29], [48].

Other pro-death (Lysine^317^) and pro-survival (Lysine^320^) acetylation sites have also been implicated to regulate p53 function and have different biological consequences [27], [30]. A significant caveat of our studies is that we only examined a couple of pro-death acetylation sites (Lysine^373^ and Lysine^382^) using the acetylated-Lysine-specific antibodies directed to the particular site of interest. It is possible that acetylation at the other known acetylation sites could change as well after cerebral ischemia and/or 17β−E_2_ treatment, but further studies will be needed to address this issue. In addition to acetylation, other post-translational modifications can also influence p53 function and could be regulated by 17β−E_2_. For instance, studies in breast cancer cells revealed that 17β−E_2_ inhibited resveratrol-stimulated phosphorylation of Serines^15, 20 and 392^ of p53 and acetylation of p53 in a concentration-and time-dependent manner, and decreased apoptosis [49]. To our knowledge, no one has examined whether 17β−E_2_ exerts a similar regulatory effect upon phosphorylation of p53 in the brain following cerebral ischemia. Further studies are thus needed to address this issue. Finally, it has been demonstrated that p53 can be methylated at Lysine^372^, which leads to enhanced stability and activity of p53 [50]. Intriguingly, we found that 17β−E_2_ treatment significantly attenuated methylation of p53 at Lysine^372^ following global cerebral ischemia, which may contribute to decreasing stability and activity of p53 and to the overall neuroprotective effect of 17β−E_2_ (*data not shown*). Our results are in agreement with the breast cancer literature, where 17β−E_2_ similarly was found to reduce p53 methylation at Lysine^372^ [51], [52].

Our study also demonstrated the ability of 17β−E_2_ to regulate the p53-induced BH3 family members, Puma and Noxa. We showed that 17β−E_2_ neuroprotective properties prevented the elevation of these factors involved in the neuronal apoptotic pathway at 24 h after reperfusion. Work from transient GCI studies measuring Puma expression in the hippocampus CA1 [25] and in the cortex of MCAO ischemic animals [25] similarly confirms Puma elevation at 24 h following ischemic reperfusion, while the breast cancer literature provides support for 17β−E_2_ downregulation of Puma transcriptional activity [53]. Overexpression of Puma has also been shown to be sufficient to induce Bax (Bcl-2-associated X protein)-dependent neuronal death in cultured neuronal cells, further supporting its pro-apoptotic role in neurons [17].

Our data also demonstrates that 17β−E_2_ treatment induces an increased interaction of p53 with the E3 ubiquitin ligase, Mdm2, which is correlated with enhanced ubiquitination and a decrease in p53 protein levels. Interestingly, p53 acetylation sites Lysine^373^ and Lysine^382^ have been shown to be the same sites for MDM2 binding [54], [55]. Consequently, acetylation of p53 at these sites has been shown to result in an inability of MDM2 to bind p53 for degradation, and thus, p53 is kept at a high level [56]. In further support of this suggestion, administration of a histone acetylase inhibitor that specifically enhances acetylation at these sites has been shown to result in a significantly *prolonged* half-life of p53 by decreasing p53 ubiquitination [54]. Previous work has shown that the acetyltransferase, CBP/p300, plays a major role in acetylating p53. Our studies showed that administration of the CBP/p300 inhibitor, curcumin, was strongly neuroprotective against GCI, which is consistent with an important role for acetylated p53 in neuronal damage and cell death following cerebral ischemia.

Finally, an additional interesting finding of our study was that the ability of 17β−E_2_ to attenuate p53 acetylation was *lost* if the 17β−E_2_ replacement was preceded by a period of long-term estrogen deprivation (LTED) (10 week ovariectomy). We previously showed that the neuroprotective effect of 17β−E_2_ in GCI is similarly lost following LTED [36]. The loss of the ability of 17β−E_2_ to attenuate acetylation of p53 in the LTED animals could potentially explain the loss of the neuroprotective effect of 17β−E_2_ reported previously. An additional intriguing finding was that sham (non-ischemic) animals showed high p53 acetylation levels in the hippocampal CA1 region following LTED. The robust acetylation of p53 in animals following LTED suggests a role for *endogenous* 17β−E_2_ to restrain p53 acetylation in the hippocampus. This is intriguing, as we previously found that LTED animals are *hypersensitive* to ischemic stress, and show *enhanced* ischemic damage to the hippocampus following GCI [36]. This agrees with previous *in vitro* findings showing that acetylation of p53 *increases* the susceptibility of cells to stress [28], [29]. Thus, the elevated p53 acetylation in sham LTED animals in our current study may provide an explanation for our previous report of enhanced susceptibility of LTED animals to ischemic stress.

In conclusion, the current study demonstrates that p53 undergoes enhanced acetylation in the hippocampal CA1 region following GCI, and that the neuroprotective hormone, 17β−E_2_, acts to strongly suppress the acetylation of p53, leading to ubiquitination and attenuation of p53 levels, as well as attenuation of the p53-regulated pro-apoptotic factors, Puma and Noxa. As a whole, the studies enhance our understanding of p53 regulation in the brain following GCI, and suggest a potentially important regulatory role for estrogen in the control of p53.

## Supporting Information

Figure S1 A) Lack of effect of estrogen on basal Acetyl p53- Lysine^373^ levels and Puma levels in sham non-ischemic control animals. Estrogen (E2) has no significant effect upon Acetyl p53- Lysine^373^, p53, and Puma levels in the hippocampal CA1 region of non-ischemic sham control animals as compared to placebo (Pla) treated animals. B) Estrogen attenuates Acetyl p53 (Lysine^382^) levels at 3 hours following ischemic reperfusion. Acetylation of p53 at Lysine^382^ did not change in Pla and E2-treated shams. A significant increase in p53 acetylation at Lysine^382^ is observed in Pla-treated animals at 3 h after ischemic reperfusion, whereas E2-treatment significantly attenuated this elevation. Total p53 levels remained unchanged in all treatment groups examined. *p <0.05 vs. sham and ^#^p <0.05 vs. Pla group.
(TIF)
