# Supplementary figures and images for: Acetylation of the Pro-Apoptotic Factor, p53 in the Hippocampus following Cerebral Ischemia and Modulation by Estrogen
Source: PLoS One. 2011 Oct 26;6(10):e27039. doi: 10.1371/journal.pone.0027039 (PMC3202599; doi:10.1371/journal.pone.0027039)

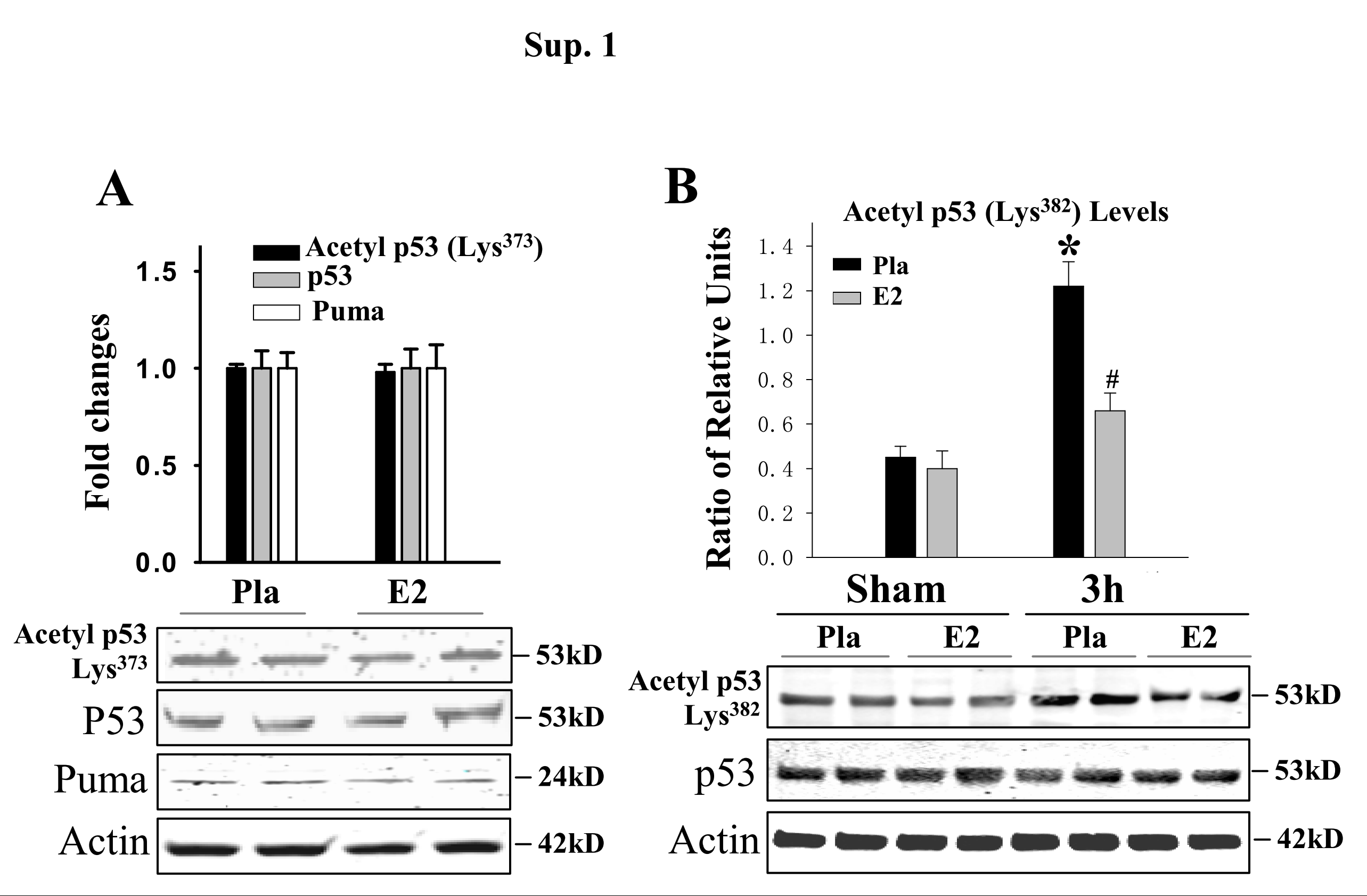

Supplement: Figure S1 — A) Lack of effect of estrogen on basal Acetyl p53- Lysine373 levels and Puma levels in sham non-ischemic control animals. Estrogen (E2) has no significant effect upon Acetyl p53- Lysine373, p53, and Puma levels in the hippocampal CA1 region of non-ischemic sham control animals as compared to placebo (Pla) treated animals. B) Estrogen attenuates Acetyl p53 (Lysine382) levels at 3 hours following ischemic reperfusion. Acetylation of p53 at Lysine382 did not change in Pla and E2-treated shams. A significant increase in p53 acetylation at Lysine382 is observed in Pla-treated animals at 3 h after ischemic reperfusion, whereas E2-treatment significantly attenuated this elevation. Total p53 levels remained unchanged in all treatment groups examined. *p <0.05 vs. sham and #p <0.05 vs. Pla group. (TIF) [file pone.0027039.s001.tif]
